# A comprehensive pediatric cardio-oncology program: a single institution approach to cardiovascular care for pediatric patients with cancer and childhood cancer survivors

**DOI:** 10.1186/s40959-024-00211-7

**Published:** 2024-04-06

**Authors:** Nathanya Baez Hernandez, Ksenya Shliakhtsitsava, Drishti Tolani, Cindy Cochran, Ryan Butts, Judith Bonifacio, Elizabeth Journey, Jenna N. Oppenheim, Sarah G. Pennant, Kimberly Arnold, Terri McCaskill, Daniel C. Bowers

**Affiliations:** 1 Department of Pediatrics, Division of Pediatric Cardiology,, University of Texas Southwestern Medical Center, Dallas, TX USA; 2 Department of Pediatrics, Division of Pediatric Hematology-Oncology, University of Texas Southwestern Medical Center, Dallas, TX USA; 3 Center for Cancer and Blood Disorders, Children’s Health, Dallas, TX USA; 4 Department of Pediatrics, Children’s Health, Dallas, TX USA; 5 Clinical Nutrition, Children’s Health, Dallas, TX USA; 6 Department of Psychology, Children’s Health, Dallas, TX USA; 7 Department of Psychiatry, UT Southwestern, Dallas, TX USA

**Keywords:** Childhood cancer survivor, Cardiovascular, Cardio-oncology, Cardiotoxicity

## Abstract

Cardiovascular complications related to cancer therapies are broad and variable in onset. These complications are the leading cause of non-cancer related morbidity and mortality in childhood cancer survivors and can also impact ongoing cancer treatment. Despite this understanding, dedicated cardio-oncology programs are lacking in pediatric cardiology. In an attempt to respond to these concerns, a risk-stratified, comprehensive cardio-oncology program was established to address the cardiovascular needs including prevention, early diagnosis, and management of patients with and at risk for cardiovascular complications of cancer therapy. This manuscript describes a single institution’s experience of building and managing a multidisciplinary pediatric cardio-oncology program with close collaboration among cardiologists, oncologists, advanced cardiology and oncology practice providers, and allied health providers such as a dietitian and psychologist to provide comprehensive cardiovascular care for childhood cancer patients and survivors. In developing this program, emphasis was on the childhood cancer survivor population, as various cardiovascular complications can present many years after cancer treatment.

## Background

Remarkable advances in diagnosis, risk stratification-based therapy, and treatment of children and adolescents with cancer over the past decades have resulted in ever increasing numbers of long-term survivors of childhood cancer. At present, the estimated 5-year survival rate for individuals under age 18 who were diagnosed with cancer between 2010 and 2016 is approaching 85% [1], which translates to currently more than 500,000 childhood cancer survivors (CCS) in the United States [2]. Despite these improvements in survival rates, cardiovascular late effects can impact both physical health and psychosocial well-being in survivorship [3–5].

Cardiovascular (CV) complications include but are not limited to cardiomyopathy, coronary artery disease, pericardial disease, valvular and vascular dysfunction. All of these may lead to heart failure and are significantly more prevalent among CCS than the general young adult population. This represents a great challenge as CV complications can potentially compromise an otherwise successful cancer treatment [4, 6, 7]. Rates of cancer-related CV disease in the form of symptomatic heart failure amongst CCS has been estimated at 4.8% by 45 years of age and asymptomatic heart failure, commonly known as subclinical cardiomyopathy, has been estimated as high as 27% [7, 8]. Furthermore, CCS have a 7-fold risk of developing CV disease when compared to the general population [9]. As a result, national and international collaborations have been developed to create risk-stratified recommendations for screening for CV dysfunction which include the Children’s Oncology Group’s *Long-Term Follow-Up Guidelines* and the *International Late Effects of Childhood Cancer Guideline Harmonization Group* [10, 11].

The increased understanding of cancer treatment-associated CV complications has led to the need for a comprehensive CV assessment of childhood cancer patients and should start at the time of cancer diagnosis with continuation throughout their lives. Despite these recognized needs, dedicated cardio-oncology services are lacking in pediatric cardiology. A survey in 2019 disseminated to pediatric cardiologists by the American College of Cardiology (ACC) working group reported that only 5% of respondents were working at an institution with a dedicated cardio-oncology specialty service [12]. Even though expert consensus guidelines for risk-stratified screening for cardiomyopathy exist, recommendations for when abnormalities are detected are less defined [12, 13]. Additionally, the limited involvement of CV assessment by cardiologists in the pre-therapy evaluation and limited systematic counseling regarding CV risk factors were demonstrated by the same survey conducted by the Pediatric Cardio-Oncology Working Group of the ACC [12]. Pediatric cardiology services are usually available in academic institutions to provide consultations to oncology services when cancer-related CV complications arise; however, models are not well established that include risk stratification with active cardiology involvement prior to, during, and after cancer treatment despite a clear necessity for standardized CV care in this patient population. At University of Texas Southwestern (UTSW) Medical Center and Children’s Health, we recognized the value of joint efforts between pediatric oncology and pediatric cardiology by providing an enhanced, comprehensive, lifelong approach to CV care for both childhood cancer patients receiving cardiotoxic therapy and CCS with prior exposure to cardiotoxic therapies. As a result, a more unified approach was established through the development of the cardio-oncology program. In this article, we describe the construction of the necessary elements for a coordinated and multidisciplinary team approach.

## Cardio-oncology Program Development and Practice

### Logistics and structure

The establishment of a cardio-oncology program required institutional clarity to reflect the ability of the leading teams to provide the care needed for this cancer population, the scope of the specialties involved, and clinical staff expertise.

Team members in pediatric cardiology and pediatric oncology met in October of 2019, with the aim of developing a comprehensive cardio-oncology program. Goals, team structure, and patient eligibility criteria were proposed and discussed. Pediatric cardiologists with expertise in cardiomyopathy and pediatric oncologists with interest and expertise in survivorship became recognized leaders of the program. Dedicated advanced practice providers (APPs) and nurses from cardiology and oncology teams, pediatric registered dietitians, pediatric psychologists, and a dedicated program coordinator were identified to become part of the program as well. To provide a framework for cardiac care of childhood cancer patients and survivors at UTSW Medical Center and Children’s Health, institutional guidelines were created. These guidelines were based upon evidence-based recommendations from the Children’s Oncology Group’s *Long-Term Follow-Up Guidelines*, International Late Effects of Childhood Cancer Guideline Harmonization Group, Heart Failure Guidelines, and supplements as necessary by clinical experience and consensus expert opinion [10, 11, 14].

Oncology clinic space was selected for outpatient multidisciplinary visits because this location was familiar to patients and families already enrolled in the well-established survivorship program named After the Cancer Experience (ACE). These multidisciplinary visits are coordinated on a monthly basis at the time of routine cancer survivorship evaluations. A road map/checklist was created for patients and their families to provide a visual flow of imaging studies, laboratory assessments, and providers they will see the day of the clinic appointments. Clinic staff received training regarding multidisciplinary encounter scheduling, and partnership with the cardiology imaging department facilitated prompt studies when needed with designated echocardiogram slots reserved for such patients.

**Fig. 1:**
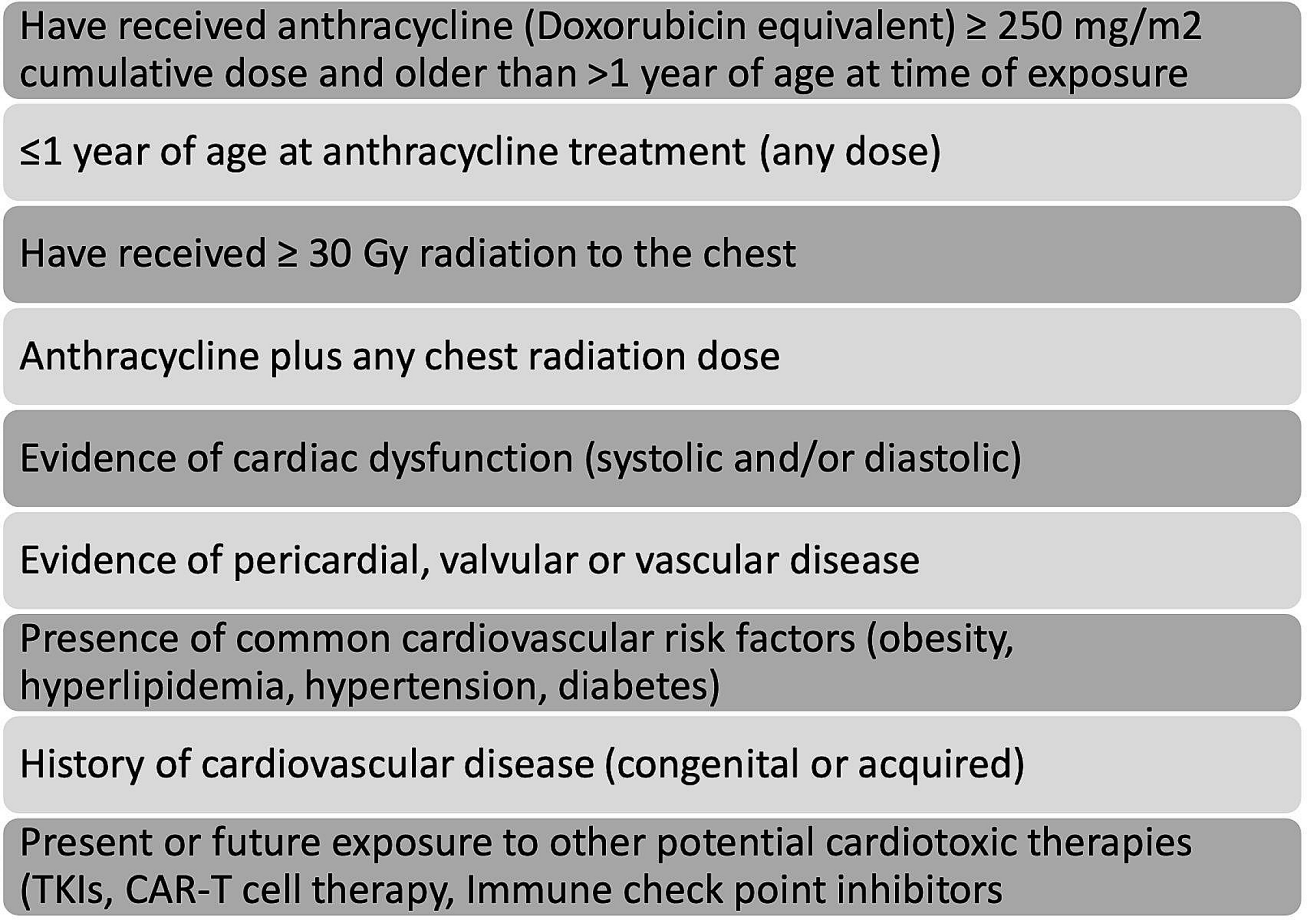
Eligibility Criteria for Outpatient Cardio-Oncology Clinic. TKIs: tyrosine kinase inhibitors, CART-T: Chimeric Antigen Receptor T Cell

Referral criteria for the outpatient cardio-oncology clinic was established (Fig. 1). For management and follow-up purposes, patients referred to the cardio-oncology clinic were classified into one of the following 3 groups based on cardiovascular risk and degree of heart disease: screening population, early intervention, and heart failure (Table 1). Further management was guided based on the heart failure guidelines [14].

**Table 1:** Medical approach in cardio-oncology program based on patient categories

Group class	Definition	Clinical approach	Visit setting
Screening population	Cancer patients and CCS at risk of cardiotoxicity due to cardiac and oncologic history but no evidence of cardiac dysfunction or symptoms	Visit focused on cardiovascular health counseling and management of CV risk factors	Cardio-oncology multidisciplinary survivorship clinic
Early intervention group	Evidence of mild subclinical cardiotoxicity based on echocardiographic or CMR parameters	Cardiovascular health counseling and management of risk factors plus consideration of cardiac medications based on HF guidelines	Cardio-oncology multidisciplinary survivorship clinic
Heart failure group	Patients with moderately or severely depressed cardiac function or symptomatic heart failure	Cardiovascular health counseling and management of risk factors plus HF guided medical therapy	Cardio-oncology heart failure clinic

CCS: childhood cancer survivor, EF: ejection fraction, GLS: global longitudinal strain, CV: cardiovascular, HF: heart failure, CMR: cardiac magnetic resonance

### Collaborative multidisciplinary team approach to patient care

**Fig. 2:**
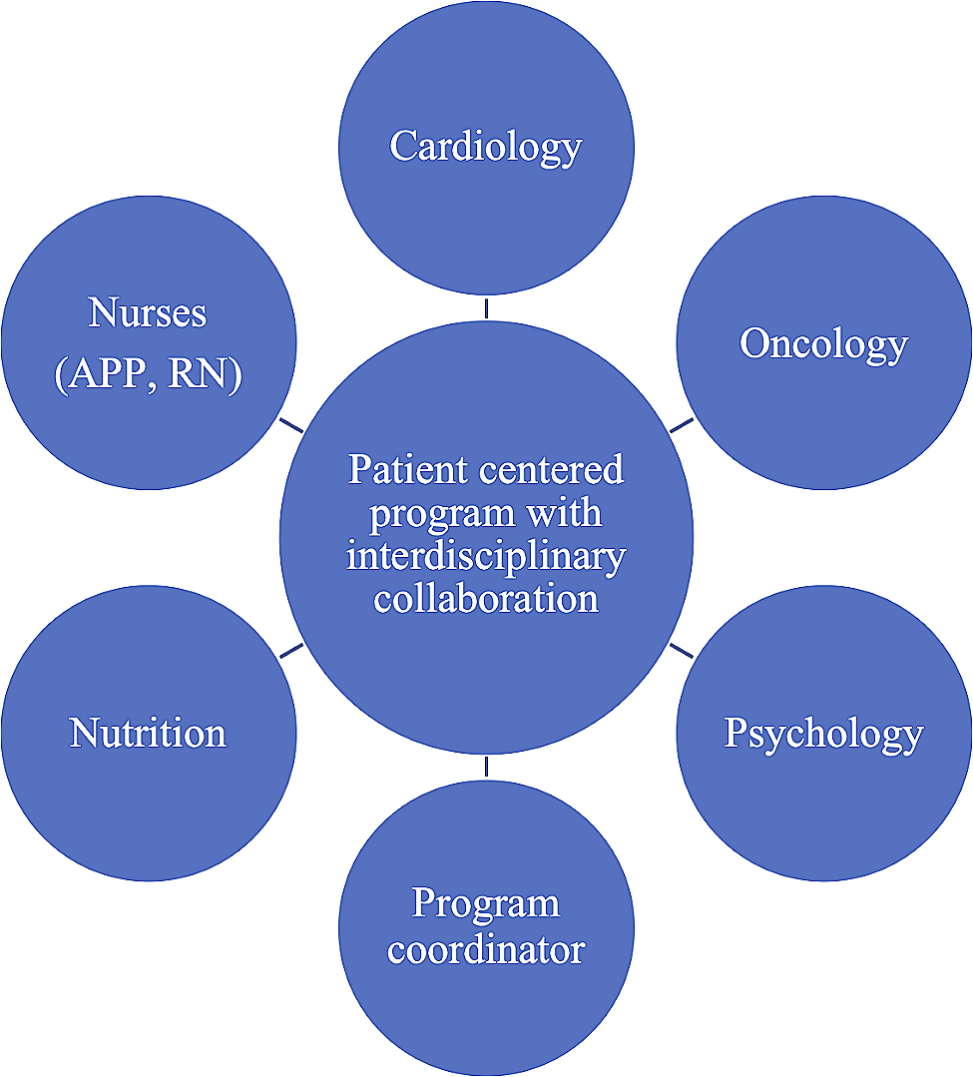
Cardio-oncology Multidisciplinary Team Structure. APP: advance practice provider, RN: registered nurse

The cardio-oncology team brings together pediatric cardiologists, pediatric oncologists, pediatric oncology and cardiology advanced practice providers (APPs), dietitians, and psychologists who have specialized clinical interests, expertise, and experience in working with children who are receiving or have completed cancer treatment. This multidisciplinary team works together to comprehensively manage all aspects of care (Fig. 2). A multidisciplinary and holistic approach is utilized to address patients’ cardio-oncology needs and ensure that families understand prevention and treatment plans. The centralized care model allows families and patients to see all providers in an efficient, single day clinic encounter. Medical decisions based upon therapeutic exposures and cardiovascular risk features are discussed in a post-clinic debriefing among the members of the multidisciplinary team.

#### Pediatric cardiology

The cardio-oncology program includes pediatric cardiologists with expertise in cardiomyopathy, heart failure and cancer-related cardiotoxicity. Pre-cancer treatment evaluation and screening is performed at the request of the oncology team based on patients’ medical history (current and/or prior cancer, anticipated cancer therapies, cardiac history, presence of CV risk factors), laboratory evaluations, and cardiac imaging findings. Inpatient and outpatient CV care is provided by the same team of cardiologists who are identified as the core team for the oncology patients. This allows for a streamlined approach for referral and ensures that continuity of care is maintained. Given the lack of cost-effective systematic screening and management guidelines of cancer related cardiac dysfunction, personalized care is provided based upon cancer therapy and patient clinical characteristics and risk features.

The cardio-oncology team is also supported by an electrophysiologist, interventional cardiologist, and cardiothoracic surgery team. Some patients may require further interventions such as advanced cardiac support in the form of a ventricular assist device or extra-corporeal membrane oxygenation for cardiac support while aiming for cardiac recovery. These decisions are made in a multidisciplinary fashion with the oncology team as the overall cancer prognosis and patient condition are relevant in decision making.

#### Pediatric oncology

The primary pediatric oncologic diseases at risk for cardiovascular complications include high-risk leukemias, lymphomas (both Hodgkin’s disease and non-Hodgkin’s lymphomas), sarcomas, and high-risk Wilms tumors. In this regard, pediatric oncologists with specific expertise in potential cardiotoxic treatments and survivorship care are essential for the proper risk stratification and management of late effects.

#### Advanced practice providers

Advanced Practice Providers who are trained in oncology and cardiology are key members of the team and collaborate with the physicians in caring for this cancer population in both the inpatient and outpatient settings. At our institution, APPs evaluate patients independently and ultimately develop strong patient-provider relationships throughout the patient’s medical journey including transitions into adult survivorship.

#### Registered dietitian nutritionist

Risk of CV disease among CCS may be increased if dietary patterns and lifestyle are not supportive of overall health [15]. As part of the multidisciplinary cardio-oncology team, evaluation by a Registered Dietitian Nutritionist (RDN) may help with amelioration and prevention of modifiable dietary risk factors. Individualized medical nutrition therapy can address these risk factors and promote therapeutic changes [16]. Our RDN provides a personalized nutritional evaluation and counseling with emphasis on fruits, vegetables, whole grains, and protein sources including fish and plant-based options. These dietary changes, as well as moderate exercise, can help lower BMI and promote a healthy weight [17].

#### Psychology

Several disease-related sequelae can impact not just the physical health but also psychosocial well-being of CCS. To this end, physicians from oncology and cardiology advocated for integration of psychological services into the new cardio-oncology clinic. A pediatric psychologist with expertise in psycho-oncology evaluates children, adolescents, and young adults during their cardio-oncology encounter and screens for psychological disorders including anxiety, depression, and neuropsychological concerns. Studies suggest that screening is even more important for high-risk survivors with serious late effects such as cardiotoxicity [18]. Patients and caregivers meet with the psychologist who conducts a brief clinical interview to identify concerns across a variety of psychosocial domains (e.g., academic, family system, peer relationships, mood, etc.), as well as complete measures assessing psychosocial health. This process promotes open communication about various psychosocial concerns and allows the psychologist to tailor the clinical interview to meet patients’ needs.

### Patient care: Outpatient Cardio-Oncology Care and Inpatient Consult Service

Cardiovascular care is the center of the cardio-oncology program thus the aim is to provide services to address these needs at different levels of care based on the patient’s CV diagnosis and risks. Indications for referral to cardio-oncology, initial evaluation components, screening tests, treatment modalities, and follow-up needs were established. Key considerations included the type of patients to be referred and evaluated and in which settings (Table 1). These were defined based on patients’ CV risks, diagnosis, and frequency of cardiac care follow-ups.

#### Outpatient care

Outpatient CV care takes place in the comprehensive multidisciplinary survivorship clinic and the cardio-oncology heart failure clinic.

The electronic medical record (EMR) embedded within the survivorship program (ACE) database is utilized to identify patients who are at high risk for CV disease (based on cancer type, treatment exposure, cardiac history and/or the presence of CV risk factors). Patients who meet inclusion criteria for cardio-oncology clinic are invited to participate in the multidisciplinary clinic. Patients at risk but without current evidence of cardiac disease (screening population) are seen in the comprehensive cardio-oncology clinic usually 2 years after completion of cancer treatment as this is usually the time when transition to survivorship takes place.

The survivorship database residing in the EMR system includes comprehensive information regarding unique exposures of each patient including cancer diagnosis, staging/risk stratification details, treatment data with cumulative chemotherapy doses, cumulative radiation therapy exposure, any prior complications, and late effects. A dedicated to the long-term follow up care (ACE program) nurse practitioner updates database data at each clinic visit and enters initial information at the time of a new referral to the long-term follow up care clinic. Physicians with expertise in cardiology and survivorship care that form part of the comprehensive cardio-oncology team screen the database for potentially eligible patients in addition to identifying potentially eligible patients during routine off therapy visits.

**Fig. 3:**
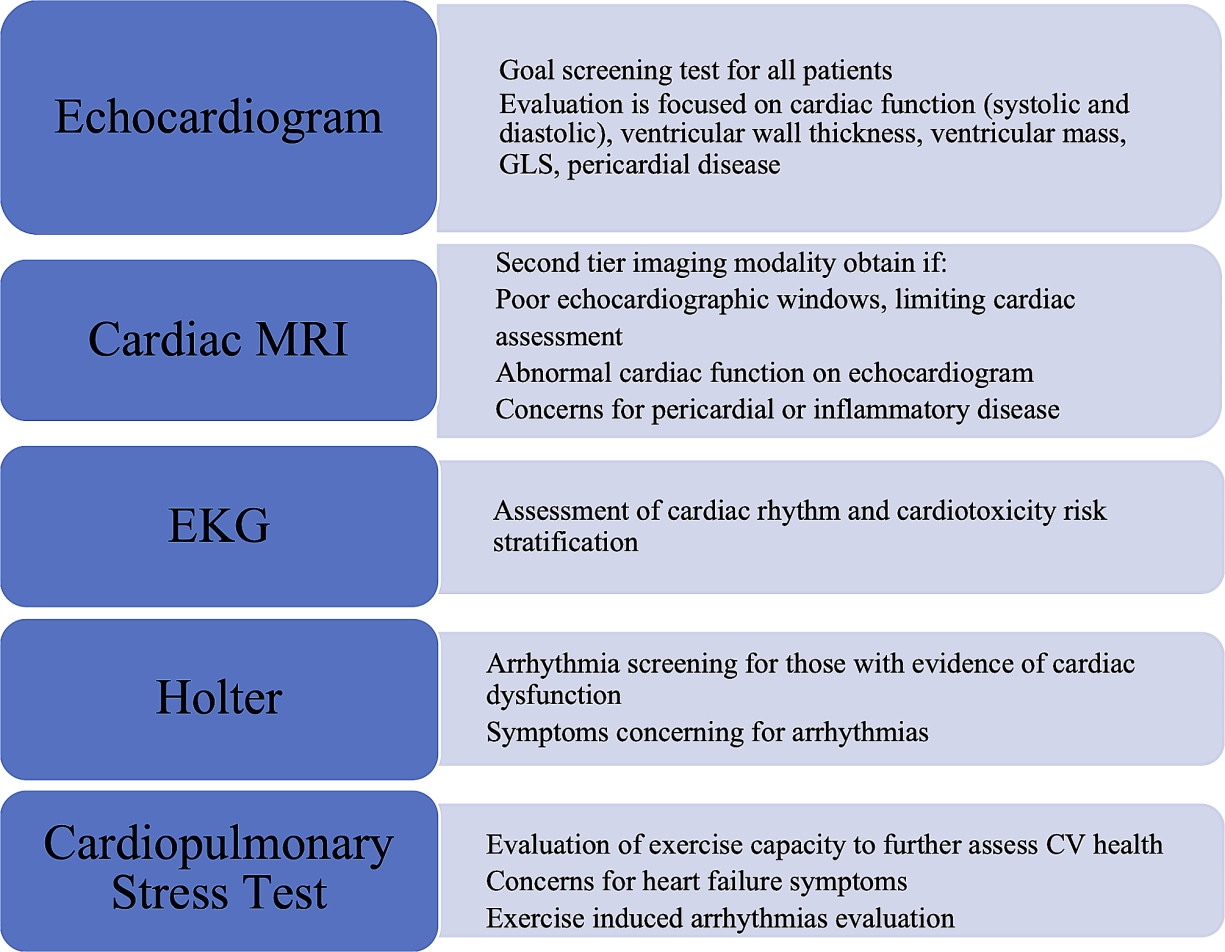
Cardiovascular Imaging Tests Modalities. MRI: magnetic resonance imaging, EKG: Electrocardiogram, global longitudinal strain, CV: cardiovascular

Necessary elements during the multidisciplinary clinic visit include blood work, cardiac imaging and cardiology, oncology, psychology, and dietitian assessments. The initial cardiac evaluation includes a thorough clinical and family history, physical examination, and laboratory and imaging studies. Echocardiography is the primary cardiac imaging screening test, and other advanced imaging studies such as cardiac MRI are performed if clinically indicated (Fig. 3). Electrocardiograms are performed at every initial cardiac visit and subsequently as clinically indicated based on symptoms, cardiac and cancer treatment history. Holter monitor is an adjuvant tool if concern for arrhythmias or if significant cardiac dysfunction (Ejection fraction < 40%). Cardiopulmonary stress tests are performed to evaluate exercise capacity or further assess CV health based on symptoms or degree of cardiac disease. Screening laboratory tests are obtained based on CV risk factors.

The cardio-oncology heart failure clinic takes place in the cardiology floor and is led by cardiologists with expertise in cardiomyopathy and heart failure. These clinics are available once a week and are embedded within the heart failure clinic. Patients are evaluated in the cardio-oncology heart failure clinic if still undergoing cancer treatment, have not yet transitioned to the survivorship clinic, have symptomatic cardiac disease, or need prompt cardiovascular risk assessment prior to starting cancer treatment.

#### Inpatient consultation

**Fig. 4:**
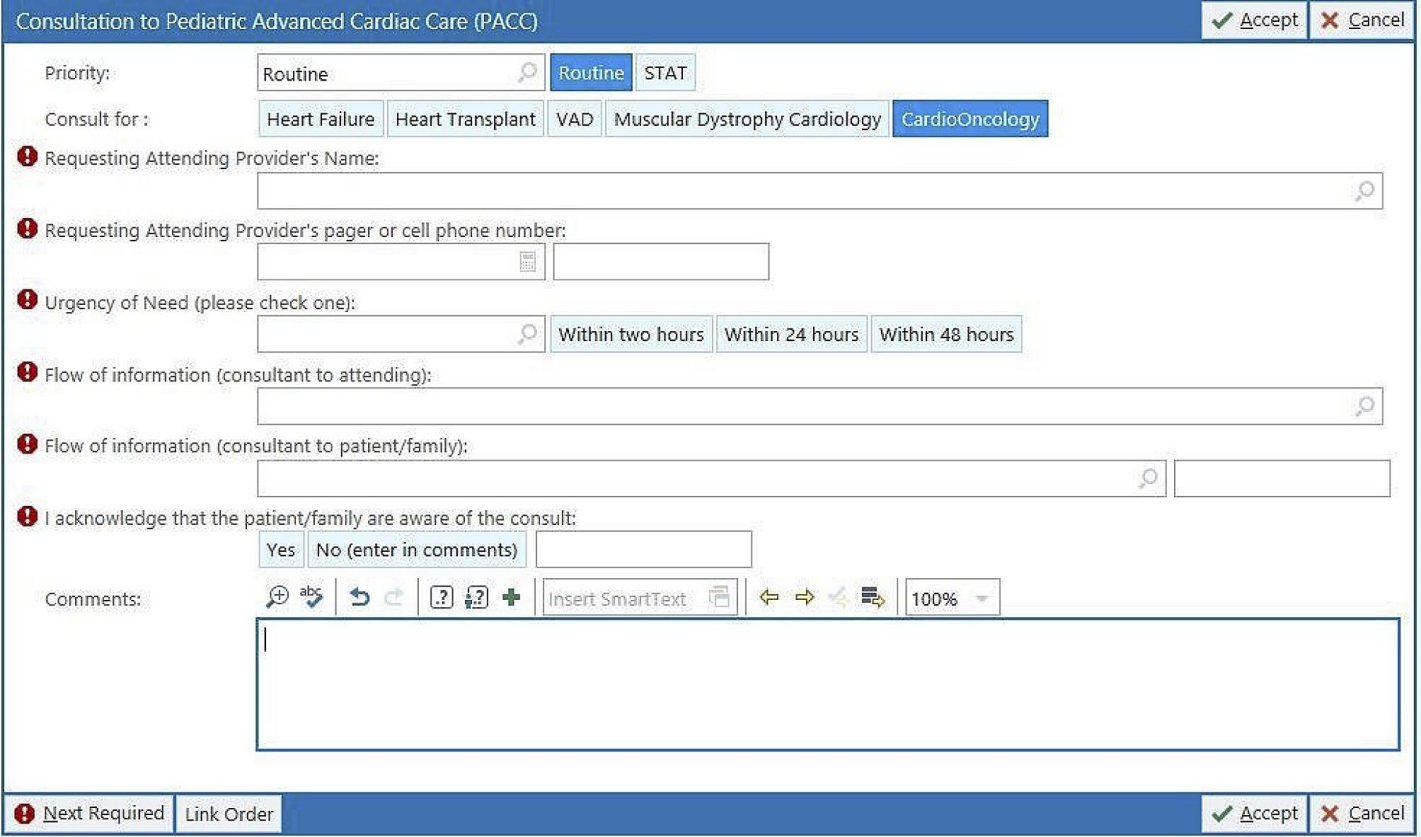
Epic) for inpatient cardio-oncology consultation request

As the awareness of the cardio-oncology program has increased within the oncology department, the recognition of streamlined consultations directed to the dedicated cardio-oncology cardiologists for the inpatient setting emerged. Subsequently, inpatient cardiology involvement at the request of the oncology team continues to improve for pediatric cancer patients. Services include newly diagnosed cardiotoxicity management, cancer treatment cardiovascular assessment and management, and recommendations for further CV testing if needed. A formal consult order set has been created in the institutional EMR to guide appropriate inpatient referrals (Fig. 4). These efforts continue to facilitate and improve continuity of cardiovascular care for cancer patients.

### Patient care: cardiac imaging

Cardiac imaging is paramount in the screening and assessment of cardiac anatomy, structure, and function during evaluation of cancer related cardiotoxicity so a strong partnership with cardiac imagers is necessary. Echocardiography and cardiac magnetic resonance imaging (CMR) protocols were established for cardiac assessment of these patients. These protocols will limit variability. Echocardiography is the most widely utilized and recommended imaging modality for initial screening of cardiac function and structural assessment as it is readily available, involves standard views that are straightforward to replicate, and involves no radiation [11].

Our cardio-oncology echocardiography protocol is in alignment with recommendations from the American Society of Echocardiography (ASE) and the European Society of Cardiology (ESC) [19, 20]. Important views to obtain include multiple two-dimensional echocardiographic measurements of left ventricular systolic function (ejection fraction measured by both Simpson Biplane and 5/6 area-length methods), left ventricular global longitudinal strain assessment to assess early changes in myocardial contractility, tissue Doppler assessment of diastolic function, and Tricuspid Annular Plane Systolic Excursion (TAPSE) assessment of right ventricular function [21]. The incorporation of strain assessment has been recognized as a valuable modality for assessment of myocardial deformation and early subtle reduction in cardiac contractility, which has been recommended by the ESC guidelines on cardio-oncology [20]. Decline in strain can predict future myocardial dysfunction, therefore, it is utilized in the diagnosis of asymptomatic cardiotoxicity [20, 22].

Cardiac magnetic resonance is another valuable imaging modality which is used as an adjuvant tool for further CV assessment [19, 20]. Pathologies such as cardiotoxicity from oncologic medications, microvascular dysfunction within the coronary arteries, myocarditis, and overt cardiac fibrosis can all occur in cardio-oncology patients over their lifetime. The assessment of T1 and T2 values, stress perfusion imaging, and presence of late gadolinium enhancement, in addition to standard functional assessment including strain, can help distinguish between these etiologies to help direct targeted cardiac medication therapy or suggest the need for alteration in the oncologic treatment plan [21, 23].

### Transition of care and partnership with adult cardio-oncology team

Among pediatric patients, the development of CV complications may not be apparent for many years post-treatment with the onset of cardiac disease occurring at or after the time of transition to adult care. Therefore, CCS are at risk of being lost to follow-up at the same time CV disease could manifest [24]. To this end, in addition to the care strategies to address traditional cardiovascular risk factors, education about the increased risk at the time of transition of care as adolescents approach adulthood is paramount to avoid survivors being lost to follow-up.

Our program is unique as it provides a continuum of care from childhood to adulthood. To further ensure successful transition, social workers are available on the pediatric and adult sides to assist with overcoming barriers to care and provide an additional layer of support during this critical period for CCS. Each patient’s transition process is highly individualized and is dependent on a variety of factors, including but not limited to risk of late effects, ongoing late effects or other complications requiring medical management, and psychosocial needs. Our team also collaborates with adult cardio-oncology providers in the Department of Family Medicine at UTSW who have expertise in survivorship care and will continue to care for our adult patients.

### Cardio-oncology program outcomes

**Fig. 5:**
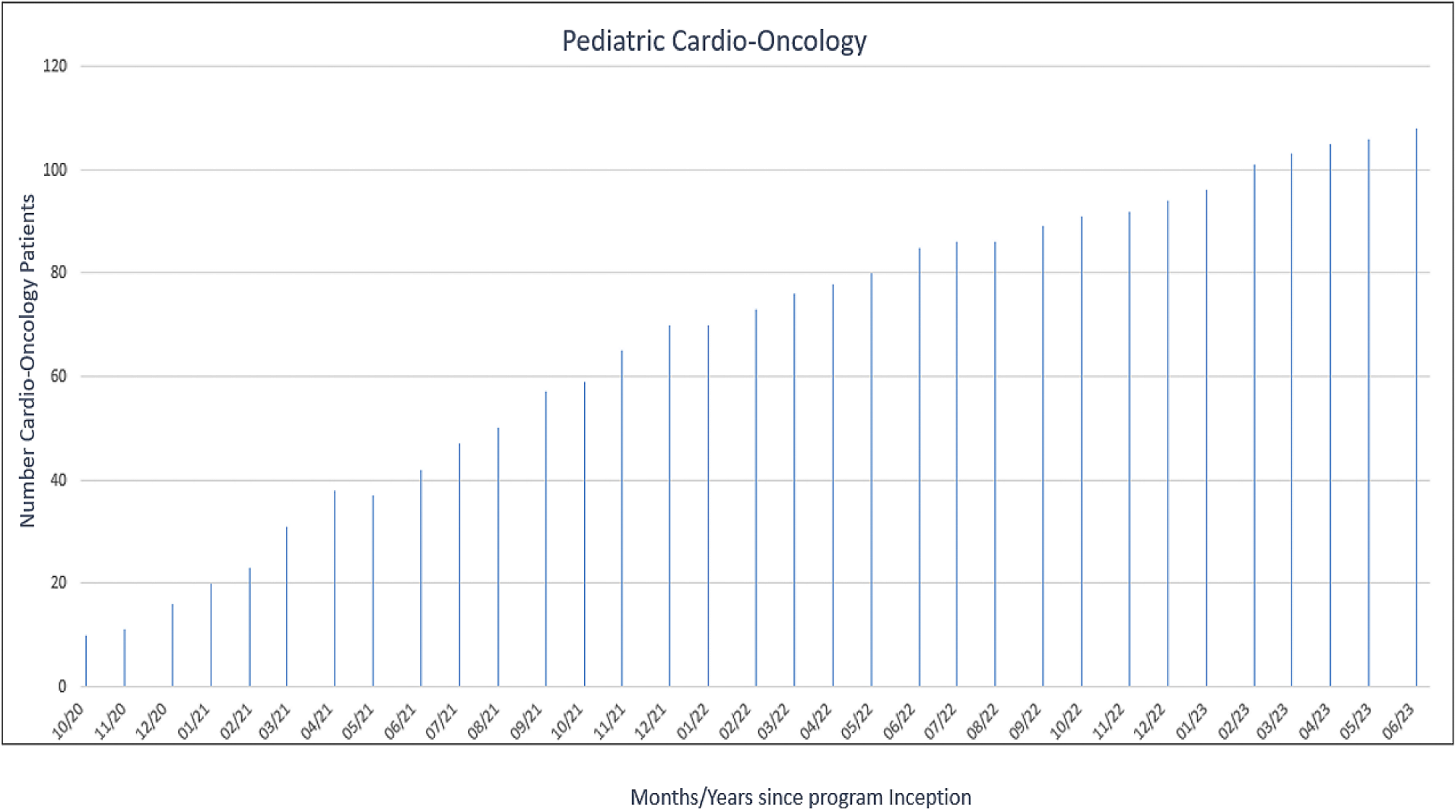
University of Texas southwestern/children’s health of Texas cardio-oncology program growth

Since its inception in October 2020, monthly cardio-oncology multidisciplinary clinic sessions have been held, and the cardio-oncology program has grown from an initial patient population of 10 to 106 patients (Fig. 5).

Seventy-eight (74%) patients are primarily followed in the outpatient multidisciplinary cardio-oncology clinic where our team utilizes a preventative approach to address cardiovascular risk factors (hypertension, hyperlipidemia, obesity) and provide screening for cardiotoxicity and treatment if indicated. Twenty-eight (26%) patients are followed primarily in the cardio-oncology heart failure clinic due to either having more advanced cardiac disease and/or still undergoing cancer treatment.

**Fig. 6:**
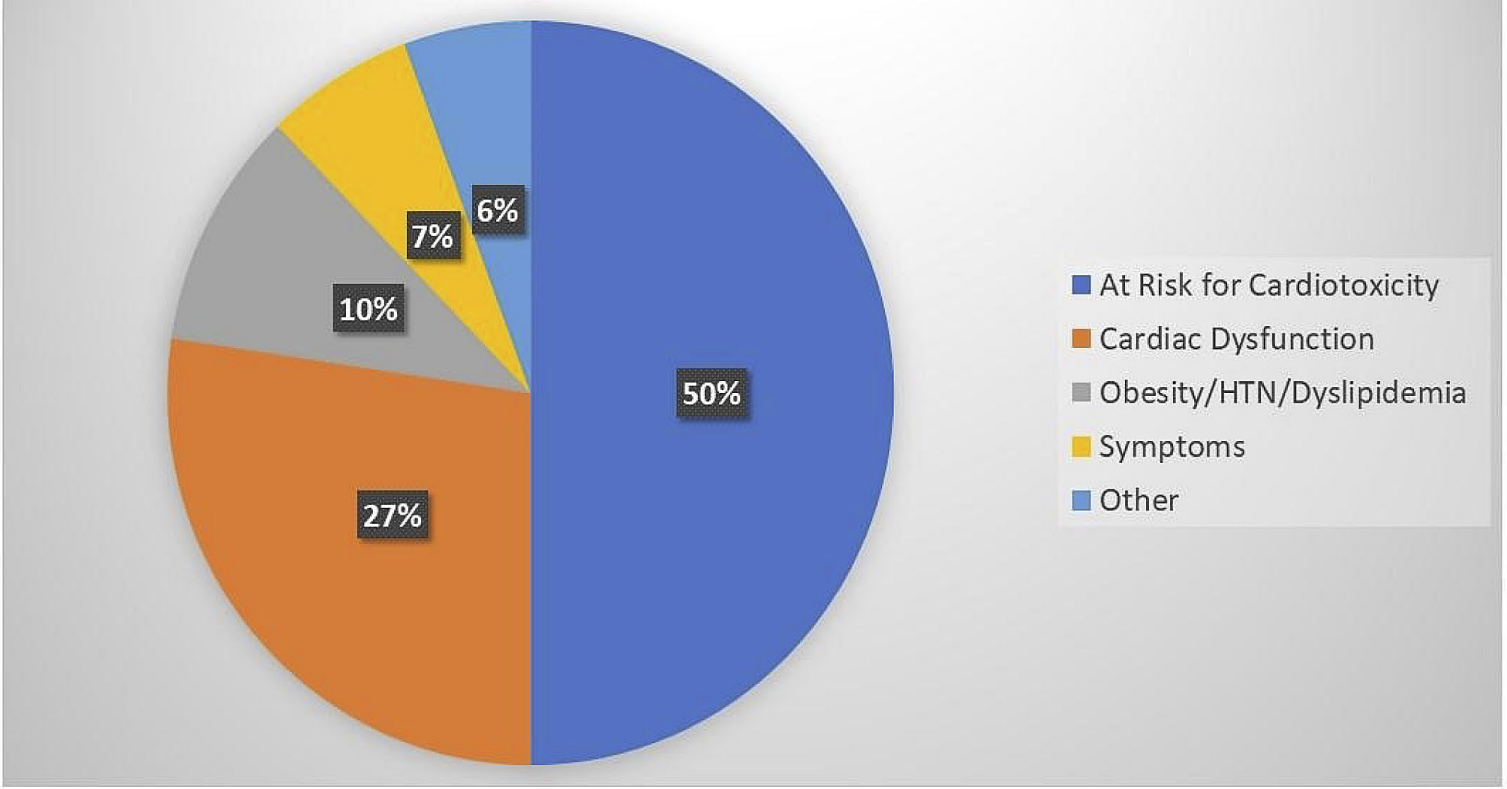
Reasons for referral to the pediatric cardio-oncology program. HTN: hypertension

**Fig. 7:**
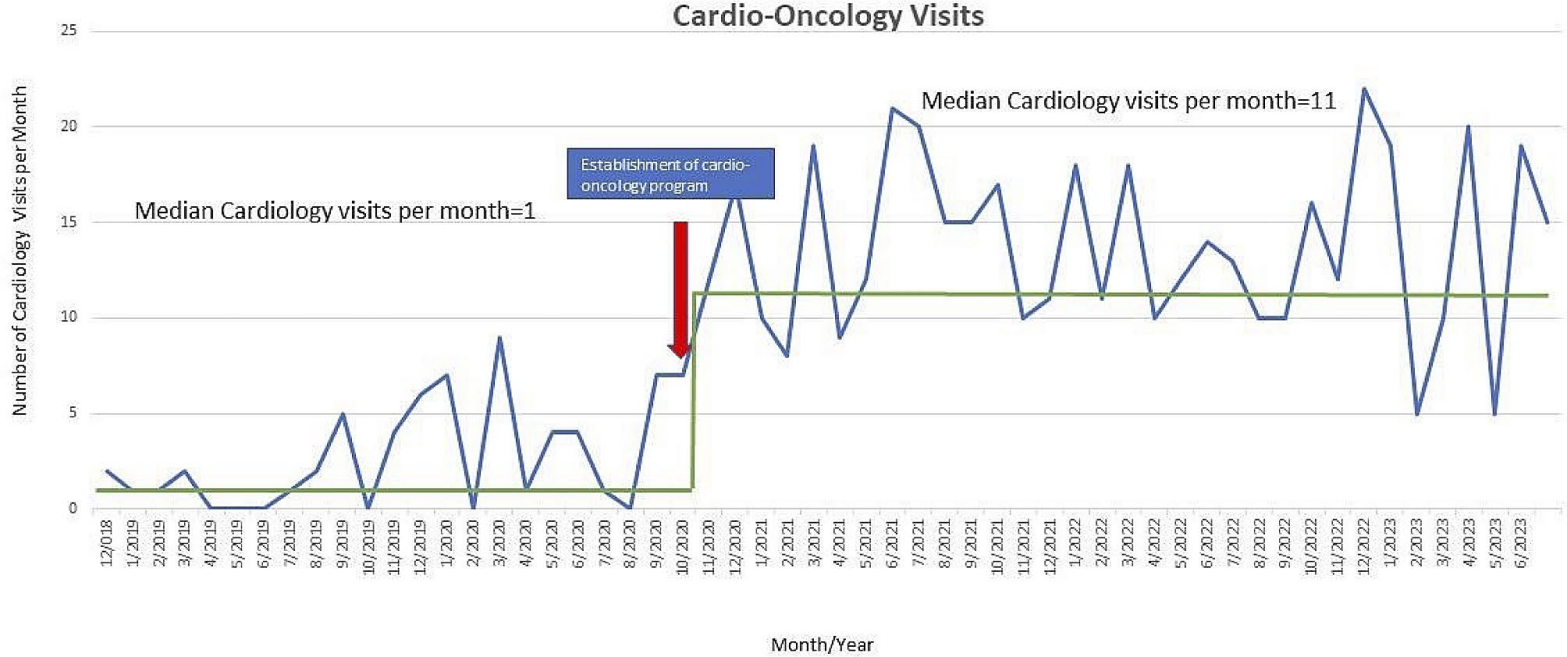
Cardio-oncology visits per month before and after program development

Reasons for patient referral to the cardio-oncology program are presented in Fig. 6. The primary reason for referral is increased risk for cardiotoxicity based on patients’ prior oncologic treatments. This is followed by evidence of cardiac dysfunction (both subclinical and clinical) being the second most common cause of referral. Seven (7%) of the patients were referred for concerns of cardiac symptoms in the form of palpitations, exertional chest pain, syncope, and fatigue. Cardiology involvement in the care of pediatric cancer patients has increased since the establishment of the cardio-oncology program with a previous median of 1 cardio-oncology visit per month increasing to 11 (Fig. 7).

**Fig. 8:**
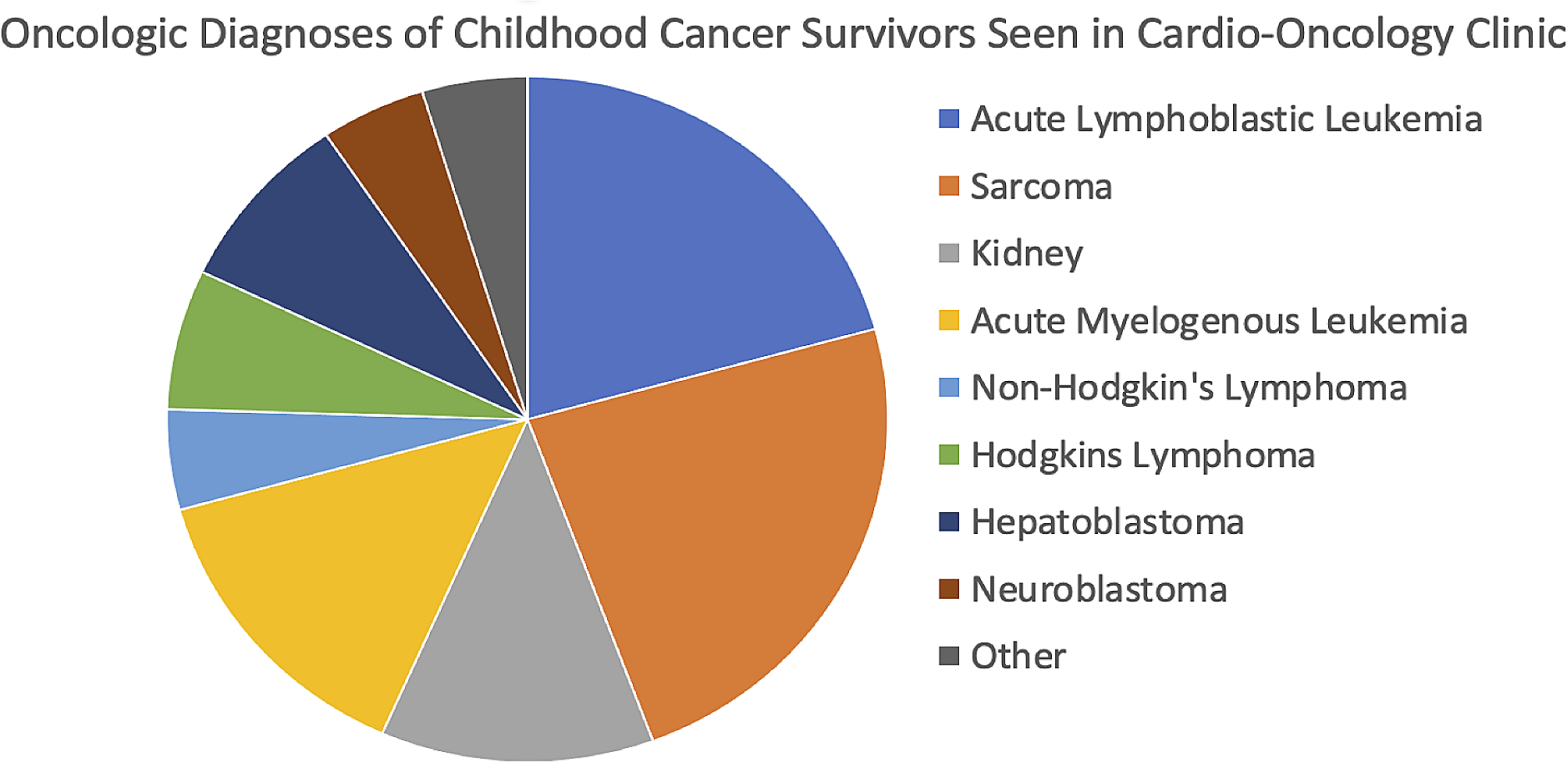
Patients’ cancer diagnoses included the following: sarcoma (24%); acute lymphoblastic leukemia (20%); acute myelogenous leukemia (14%), Hodgkin’s lymphoma (7%); non-Hodgkin’s lymphoma (5%), kidney tumors (12%); hepatoblastoma (8%), neuroblastoma (5%); and other diagnoses (5%)

Details of the demographic and clinical characteristics of the patients currently served by the cardio-oncology service are outlined in Table 2. The mean age of patients currently being seen in the cardio-oncology clinic is 15.16 years (± 4.14 years) with 62 (58%) being male. Cancer diagnosis of patients followed in the cardio-oncology program are represented in Fig. 8.

**Table 2:** Demographic and clinical characteristics of patients followed in cardio-oncology program at University of Texas Southwestern/ Children’s Health of Texas (*N* = 106)

Characteristic	*N* (%) *N* = 106
Current age (years), Mean [SD]	15.16 [4.14]
Current age (y)	
1–5	3(3)
6–10	14(13)
11–15	32(30)
≥ 16	57(54)
Male gender	62(58)
Race	
White	80(76)
Black or African American	12(11)
Other	14(13)
Hispanic ethnicity	50(47%)
Age at cancer diagnosis (years), Mean [SD]	7 [5]
Age at cancer diagnosis (y)	
1–5	51(48)
6–10	29 (27)
11–15	23(22)
≥ 16	3(3)
Years since diagnosis	
1–2	12 (11.3)
3–5	14(13.2)
>5	80(75)
Grouped cancer diagnosis:	
Solid tumors	67(63)
Hematological malignancies	39(37)
Cumulative anthracycline dose (mg/m^2^), Mean [SD]	263 [124]
Cumulative anthracycline dose ≥ 250 mg/m^2^	54(51)
Exposure to chest radiation	23(22)
Exposure to chest radiation and anthracycline	23(22)
Comorbid medical conditions	
Obesity	28(26)
Hypertension	15(14)
Dyslipidemia	5(4.7)

Potentially modifiable CV risk factors in the form of hypertension, obesity, and hyperlipidemia are present in 48 (45%) of patients.

Cardiomyopathy (sub-clinical and clinical) is present in 30 (28%) of the patients. Most of these patients have subclinical cardiomyopathy based on strain abnormality and relaxation impairment on echocardiography with 6 (5.7%) having an ejection fraction of < 55%. However, 29 (27%) had a history of decreased cardiac function based on shortening fraction and/or ejection fraction echocardiographic parameters when referred to our program. This highlights the importance of a dedicated multidisciplinary clinic in identifying previously undetected early stages of chemotherapy-induced ventricular dysfunction and allowing implementation of prompt treatment when needed.

Our team is invested in providing excellent care to our patients and monitoring the quality of our program continuously. A program quality survey comprised of 10 questions is offered to all families during their cardio-oncology clinic visits as a platform for valuable feedback. Since program establishment, we had 42 participants in the cardio-oncology multidisciplinary clinic complete our survey (40 on paper and 2 via SurveyMonkey online). Of these, most participants (> 90%) reported that their experience in cardio-oncology clinic was a valuable one.

In addition to clinical care, education and research are essential parts of the program. Educational conferences in the form of grand rounds and educational lectures to oncology fellows and faculty are provided. The current cardio-oncology partnership at our institution has fostered the initiative of combined research projects not only in the pediatric setting, but also in adult cardio-oncology as our partnership continues to strengthen.

## Discussion

Cardiovascular complications (predominantly cardiomyopathy) are a leading cause of late morbidity and mortality in cancer survivors [9]. In most cases, cardiomyopathy presents as asymptomatic disease with subtle changes in myocardial structure and performance before evolving into changes in ejection fraction at which point the cardiomyopathy course may be too late for reversal [25]. Therefore, early detection of cardiomyopathy and timely management could potentially prevent advance of cardiac dysfunction and progression to heart failure. Risk factors for cardiotoxicity in cancer survivors have been elucidated, including higher anthracycline doses, radiation therapy involving cardiac structures, younger age at diagnosis, female sex, underlying cardiovascular disease, and other common predispositions for cardiovascular disease [4]. Recognition of these allows for risk stratification to guide a preventive clinical approach.

The field of cardio-oncology has emerged in response to address the CV health needs of cancer patients and the growing number of cancer survivors. Multidisciplinary teams have been developed at many cancer centers (especially adult centers) and cardio-oncology clinics have been established with collaboration among cardiology and oncology teams [26–28]. However, dedicated pediatric cardio-oncology programs are less well established and cardiology services are typically only provided when cardiac abnormalities are present [12]. To improve clinical outcomes and provide comprehensive, standardized CV care for childhood cancer patients and CCS, we successfully developed a dedicated multidisciplinary cardio-oncology program to meet the CV needs of pediatric and young adult patients diagnosed with cancer.

Establishing a cardio-oncology program requires institutional support and collaboration among cardiologists and oncologists on all aspects of the program’s development given how many elements are necessary. Defining the needs, outlining the scope of practice, and identifying dedicated and qualified personnel are among the first steps in establishing a program [29]. Maintaining and growing the program involves commitment to patient care, education, continued collaboration and support among the oncology, cardiology, and imaging departments.

While the cardio-oncology program at our institution is led by pediatric cardiologists and oncologists, the inclusion of APPs is also vital in the CV care of cancer patients [28]. Furthermore, during early establishment of the program, we identified core professionals in the fields of nutrition and psychology since their involvement could also improve the care for cancer patients.

Pediatric cancer survivors benefit from nutritional evaluation and counseling, specifically if their treatment modalities result in increased risk of CV disease [16]. Therefore, as part of the multidisciplinary team, a RDN provides personalized nutrition counseling that focuses on addressing modifiable cardiovascular risk factors including obesity, high blood pressure, hypercholesterolemia, hypertriglyceridemia, and diabetes mellitus [15, 16]. Incremental changes toward a nutrient-dense diet and more active lifestyle should be celebrated and encouraged in a positive environment [18].

Research demonstrates that a subset of CCS (15–20%) experience notable depression, anxiety, post-traumatic stress, suicidality, risky health behaviors, and other psychosocial distress [18, 30, 31]. The risk of poor psychosocial outcomes is higher based on patients’ cancer type [e.g., central nervous system (CNS) tumors], treatment approach (e.g., CNS irradiation, anthracyclines, hematopoietic stem cell transplant), and pre-morbid learning disorders and/or mental health diagnoses [30, 32]. Psychosocial challenges may impact survivors’ engagement in long-term follow-up. However, if patients do attend a survivorship program, they tend to have improved overall psychosocial adjustment which in turn promotes a higher chance of future attendance. Further, CCS with self-reported increased stress and distress at long-term follow-up appointments (> 5 years post-diagnosis) have been found to experience more adverse cardiovascular outcomes [33]. Although psychosocial follow-up has been identified as a standard of care for pediatric cancer survivors, practical guidelines and implementation of routine screening procedures are less clear [30, 34]. Several studies have concluded that improved delivery of psychosocial screening is associated with an integrated care model, rather than models where patients are referred out to additional specialties for an initial assessment [31, 35].

Coordinated team efforts to enhance the patient experience and streamline care are paramount in developing and maintaining a cardio-oncology program. Even though it is too early to determine how this combined effort will eventually improve outcomes, the development of this program has led to earlier detection of cardiotoxicity, improved communication, and a strong partnership between specialties.

### Future directions

It is anticipated that the program will experience further growth with the goal to improve the CV health, CV health knowledge, and overall quality of life and care among high-risk CCS. We hope the partnership established among cardiologists and oncologists will help minimize and ideally eliminate CV disease as a barrier to successful cancer treatment for pediatric cancer patients and survivors. Research including prospective, investigator-initiated studies of the pathophysiology, diagnostics, and therapies for CV effects are paramount and a calling in cardio-oncology. The establishment of our program and subsequent growth should also facilitate investigator-initiated research into the causes and treatment of cardiovascular early and late effects. Scientific collaboration among oncologists and cardiologists at a multi-institutional level for future investigations on CV disease with this group of patients is necessary because it can drive high value care. Furthermore, there is ongoing exploration of the role of data registries to inform and improve care in pediatric cardio-oncology.

## Conclusions

An institutional, multidisciplinary pediatric cardio-oncology program for childhood cancer patients and survivors has been successfully established to address the CV health needs of this high-risk growing population. The multidisciplinary pediatric cardio-oncology program has achieved substantial growth since its inception in October 2020. It is anticipated that the program will experience further growth and strive to improve the cardiovascular health of children.

## Data Availability

No datasets were generated or analysed during the current study.

## Abbreviations

CCS: Childhood cancer survivor
CV: Cardiovascular
ACC: American college of Cardiology
UTSW: University of Texas Southwestern
APPs: Advanced practice providers
RDN: Register dietitian nutritionist
EMR: Electronic medical record
CMR: Cardiac magnetic resonance
